# Metabolomics and Transcriptomics Identify Multiple Downstream Targets of *Paraburkholderia phymatum* σ^54^ During Symbiosis with *Phaseolus vulgaris*

**DOI:** 10.3390/ijms19041049

**Published:** 2018-04-01

**Authors:** Martina Lardi, Yilei Liu, Gaetano Giudice, Christian H. Ahrens, Nicola Zamboni, Gabriella Pessi

**Affiliations:** 1 Department of Plant and Microbial Biology, University of Zurich, CH-8057 Zurich, Switzerland; martina.lardi@uzh.ch (M.L.); yilei.liu@botinst.uzh.ch (Y.L.); agiud@hotmail.it (G.G.); 2 Agroscope, Research Group Molecular Diagnostics, Genomics and Bioinformatics & Swiss Institute of Bioinformatics (SIB), CH-8820 Wädenswil, Switzerland; christian.ahrens@agroscope.admin.ch; 3 Institute of Molecular Systems Biology, ETH Zurich, CH-8093 Zurich, Switzerland; zamboni@imsb.biol.ethz.ch

**Keywords:** rhizobia, papilionoid, legumes, *rpoN*, sigma factor, metabolome, RNA-sequencing, nitrogen fixation

## Abstract

RpoN (or σ^54^) is the key sigma factor for the regulation of transcription of nitrogen fixation genes in diazotrophic bacteria, which include α- and β-rhizobia. Our previous studies showed that an *rpoN* mutant of the β-rhizobial strain *Paraburkholderia phymatum* STM815^T^ formed root nodules on *Phaseolus vulgaris* cv. Negro jamapa, which were unable to reduce atmospheric nitrogen into ammonia. In an effort to further characterize the RpoN regulon of *P. phymatum*, transcriptomics was combined with a powerful metabolomics approach. The metabolome of *P. vulgaris* root nodules infected by a *P. phymatum* *rpoN* Fix^−^ mutant revealed statistically significant metabolic changes compared to wild-type Fix^+^ nodules, including reduced amounts of chorismate and elevated levels of flavonoids. A transcriptome analysis on Fix^−^ and Fix^+^ nodules—combined with a search for RpoN binding sequences in promoter regions of regulated genes—confirmed the expected control of σ^54^ on nitrogen fixation genes in nodules. The transcriptomic data also allowed us to identify additional target genes, whose differential expression was able to explain the observed metabolite changes in numerous cases. Moreover, the genes encoding the two-component regulatory system NtrBC were downregulated in root nodules induced by the *rpoN* mutant, and contained a putative RpoN binding motif in their promoter region, suggesting direct regulation. The construction and characterization of an *ntrB* mutant strain revealed impaired nitrogen assimilation in free-living conditions, as well as a noticeable symbiotic phenotype, as fewer but heavier nodules were formed on *P. vulgaris* roots.

## 1. Introduction

*Paraburkholderia phymatum* STM815^T^ is a nitrogen-fixing soil bacterium that belongs to the β-proteobacteria (β-rhizobia); it is able to induce nodules on the roots of Mimosoid and Papilionoid legumes [1]. While it had first been reported as a symbiont of the *Mimosa* spp. native to South America [2,3], it was later demonstrated to be a highly promiscuous nodulator of several Mimosoid genera [4,5], as well as some Papilionoid legumes [6,7].

Nitrogen is an essential component of all amino acids and nucleic acids. It is therefore considered to be a key limiting factor for plant growth and development. The ability of legumes to form a symbiotic association with nitrogen-fixing rhizobia gives the legumes a clear advantage over other plant species. Rhizobia are soil bacteria, able to respond to specific flavonoid compounds secreted by the roots of compatible legume plants. Subsequently, they colonize the plants’ roots, which ultimately leads to the formation of specialized root organs called nodules. Inside root nodules, the differentiated bacteroids reduce atmospheric nitrogen into ammonia, which the plant then either converts to glutamine and asparagine in indeterminate nodules, or to ureides in determinate nodules [8,9,10]. The molecular mechanisms underlying the physiological adaptation of rhizobia in the root nodules have so far been mainly studied in α-rhizobial symbiosis model systems. Several studies have relied on functional genomics technologies like transcriptomics, proteomics and metabolomics, and comparative genomics [11,12,13,14,15,16,17,18,19,20].

Compared to α-rhizobial species, very little is known about the regulation of genes required for symbiotic nitrogen fixation in β-rhizobial strains. In a previous study, we mutated the *P. phymatum* RpoN encoding gene, Bphy_0326, which exhibited the highest amino acid homology to the α-rhizobial RpoN required to activate the expression of the *nif* cluster in microaerobic conditions [21,22]. The *P. phymatum rpoN* mutant strain was affected in the utilization of urea and nitrate, and was not able to reduce nitrogen inside *P. vulgaris* nodules [23]. Thus, similar to the situation in α-rhizobia, the alternative RNA polymerase sigma factor RpoN (or σ^54^) and its enhancer binding protein (EBP) NifA are essential for nitrogenase activity during this β-rhizobial symbiosis. RpoN was originally found to be the key regulator of nitrogen utilization systems in several bacteria [24,25,26,27]. It has since been linked with several phenotypes, including motility [22], exopolysaccharides (EPS) production [27,28], biofilm formation, and other symbiotic and virulence traits [29,30,31]. This alternative sigma factor, which requires an EBP to activate gene expression, recognizes and binds consensus sequences located at the −24/−12 positions in the promoter region of target genes [32].

Here, we aimed to further characterize the RpoN regulon during symbiosis with *P. vulgaris*, using both transcriptomic (RNA-seq) and metabolomics approaches, the latter offering the option to directly investigate changes in bacteroid and plant physiology inside root nodules. For this, we extracted polar metabolites and RNA from nodules induced by both a *rpoN* (Fix^−^) mutant and from wild-type (Fix^+^) nodules. As a baseline, metabolites were extracted from uninfected roots. Pathway enrichment analyses on the detected metabolites showed that flavonoids and nucleotide precursors accumulated to higher levels in nodules induced by the Fix^−^ mutant, while the amounts of important amino acid precursors (e.g., chorismate) decreased compared to Fix^+^ nodules. Integration of the transcriptomics data from Fix^−^ and Fix^+^ nodules allowed us to explain part of the metabolic differences. Putative direct target genes of this sigma factor were identified by a genome-wide search for RpoN binding boxes in *P. phymatum* promoter regions. Finally, we constructed and characterized a *P. phymatum* mutant in the gene *ntrB*, which encodes a sensor kinase that is part of the two-component regulatory system NtrBC, and which had displayed RpoN-dependent expression inside nodules and harbored a RpoN binding sequence in the promoter region. Besides confirming the role of *P. phymatum* NtrB in nitrogen assimilation under free-living conditions, we here show that a *ntrB* mutant formed fewer but heavier nodules on *P. vulgaris* roots.

## 2. Results and Discussion

### 2.1. Metabolomic Analysis of P. vulgaris Root Nodules Infected with P. phymatum STM815^T^ Wild-Type and with a rpoN Mutant

**Figure 1:**
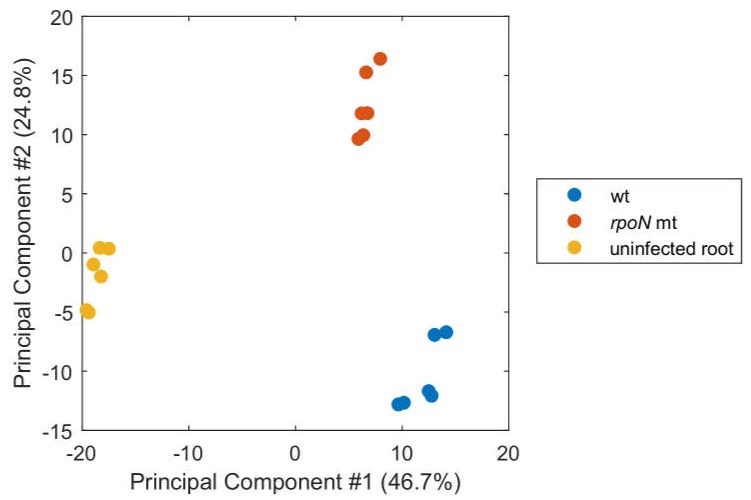
Principal component analysis (PCA) of metabolome datasets from *P. vulgaris* root nodules induced by wild-type *P. phymatum* (wt, blue) and *rpoN* mutant (*rpoN* mt, red) strains, or from uninfected *P. vulgaris* roots (yellow). Three biological replicates were analyzed, each injected twice by non-targeted metabolomics; #: number.

We have previously shown that the alternative sigma factor RpoN (σ^54^) of *P. phymatum* STM815^T^ is required for the development of an effective symbiotic interaction between this β-rhizobial strain and the legume *P. vulgaris* [23]. Although the *rpoN* mutant formed a similar number and size of nodules on *P. vulgaris* compared to the wild type, bacteroids were unable to convert atmospheric dinitrogen to ammonium. Furthermore, the nitrogen content of the shoot was significantly reduced compared to plants infected by the wild type. To gain a better understanding of the molecules and metabolic pathways relevant during the *P. vulgaris*–*P. phymatum* symbiosis, we carried out two experiments: we first compared the metabolite levels measured in *P. vulgaris* nodules infected with *P. phymatum* wild-type with levels in nodules induced by a *rpoN* mutant strain 21 days post-infection (dpi). In addition, in order to establish a metabolic baseline, we compared the metabolite levels in Fix^+^ nodules with those from uninfected root material. Three independent biological replicates were prepared, and aliquots from each replicate were injected twice (as technical replicates) in a 6550 iFunnel Q-TOF mass spectrometer (see Section 3. Materials and Methods) [33]. After discarding unassigned metabolites, low-abundant signals and adducts, a total of 409 ions, corresponding to known deprotonated metabolites from bacterial or plant origin, were identified. A principal component analysis (PCA) separated the different biological samples, according to the presence or absence of the symbiont (in the first dimension) and to the genetic background of the two strains (wt versus *rpoN* mutant, in the second dimension) (Figure 1).

To discover general RpoN-dependent metabolic trends, a metabolite set enrichment analysis for both significantly decreased and increased metabolites was performed, which identified several affected metabolic pathways (Table 1).

A comparison of ion intensities revealed that 147 (36%) of all detected and assigned ions were differentially accumulated in nodules induced by the wild-type strain (wt nod) versus those by the *rpoN* mutant (*rpoN* mt nod) (increase/decrease of more than 1.4-fold change is equivalent to a log_2_ (FC) of 0.5, and *q*-value < 0.01; see Section 3. Materials and Methods,) (Table 2). Among these, 70 metabolite ions showed a significant decrease in nodules infected by the *rpoN* mutant, while 77 had significantly increased intensities. One example of an over-represented category is the flavonoid/isoflavonoid biosynthesis pathway, which covered 13 of the 77 compounds that were more abundant in Fix^−^ nodules (Table 2). Flavonoids are phenolic compounds, which are secreted by plants in order to attract rhizobia to the root. In response to flavonoids, rhizobia activate *nod* (nodulation) gene expression, leading to the synthesis of lipochitooligosaccharides, also known as Nod factors. Besides this well-studied role, flavonoids have multiple functions, including a role as an antioxidant [34] and as a defense mechanism against pathogens [35]. The *rpoN* Fix^−^ mutant may therefore be recognized as a cheater by *P. vulgaris*, which accumulates flavonoids such as naringenin to defend itself. Naringenin has been detected in root exudates of a 9-day-old *P. vulgaris* seedling, and has been shown to induce expression of *nod* genes in *Rhizobium leguminosarum* [36]. A similar stress situation was observed in soybean nodules induced by the *Bradyrhizobium diazoefficiens* Fix^−^ regulatory mutant *nifA*, where a phytoalexin was accumulating as a defense reaction [16,37]. Other examples of pathways over-represented among metabolites accumulating in Fix^−^ nodules included the citrate cycle (TCA), as well as the connected glyoxylate and dicarboxylate pathways. In fact, isocitrate, aconitate, oxalate, and tartaric acid accumulated inside Fix^−^ nodules (Table 2). The amino acid lysine and the metabolites involved in its biosynthesis (diaminopimelate) and degradation (pipecolate) were found in the list of compounds with reduced amounts in Fix^−^ nodules (Table 1 and Table 2). Several acyl donors involved in fatty acid elongation (octanoyl-CoA, tetradecanoyl-CoA, (*S*)-3-hydroxytetradecanoyl–CoA and 3-hydroxy-5-methylhex-4-enoyl-CoA) also showed decreased amounts in Fix^−^ nodules. The ion corresponding to chorismate was the metabolite with the highest decrease in Fix^−^ nodules, compared to wild-type nodules. Chorismate is a precursor for the aromatic amino acids (phenylalanine, tryptophan, and tyrosine), indole derivatives, salicylic acid, alkaloids, vitamin K, and folate. Interestingly, phenylalanine is transformed into 4-coumaroyl-CoA, which is a precursor for flavonoid biosynthesis [38]. Therefore, the decrease in chorismate levels is in line with the accumulation of flavonoids/isoflavonoids in Fix^−^ nodules. While the glutamine level was much lower in nodules occupied by the *rpoN* mutant than in wild-type nodules, glutamate was more abundant, suggesting that RpoN controls nitrogen assimilation inside the nodule.

We also compared the abundance of metabolites detected in wild type-infected *P. vulgaris* nodules with those from the roots of uninfected plants (baseline): 169 compounds showed increased abundance in nodules, while 62 accumulated in the roots (Table S1). Interestingly, out of the 70 metabolites with significantly decreased abundance in nodules infected by the *rpoN* mutant (s. above), 63 (90%) were also found among the 169 metabolites accumulating in the nodules compared to the roots. This suggested an important role of *P. phymatum* RpoN in *P. vulgaris* nodule physiology (Figure S1). For instance, chorismate was the metabolite decreasing the most in Fix^−^ nodules, compared to Fix^+^ nodules, and the compound that accumulated the most in nodules compared to roots. Examples of compounds following the same pattern of a decrease in Fix^−^ nodules compared to Fix^+^ nodules were glutamine, arginine, alanine, and lysine. In contrast, only 17 out of the 77 metabolites accumulated in nodules occupied by the *rpoN* mutant displayed increased amounts in the roots, compared to wild-type nodules (Figure S2). This result suggested that *P. vulgaris* reacted to the presence of the *P. phymatum* Fix^−^ mutant with a specific reaction, including the accumulation of flavonoids/isoflavonoids, nucleotides, and nucleotide sugars.

Very little is known about the nutrients (carbon and nitrogen sources) consumed by the β-rhizobial strain *P. phymatum* during symbiosis with legumes. To mimic the root nodule environment, we performed a Biolog experiment by growing *P. phymatum* wild-type cells under micro-oxic conditions, in 96 well plates with different carbon (C) or nitrogen (N) sources. A list of the compounds used by *P. phymatum* under micro-oxic conditions is provided in Table S2. Interestingly, several compounds that were more abundant in nodules compared to roots (Table S1), such as glutamine and arginine, were utilized well by *P. phymatum* as C and N sources (Table S2).

### 2.2. RpoN Regulon in Symbiosis, as Determined by Transcriptomics

**Figure 2:**
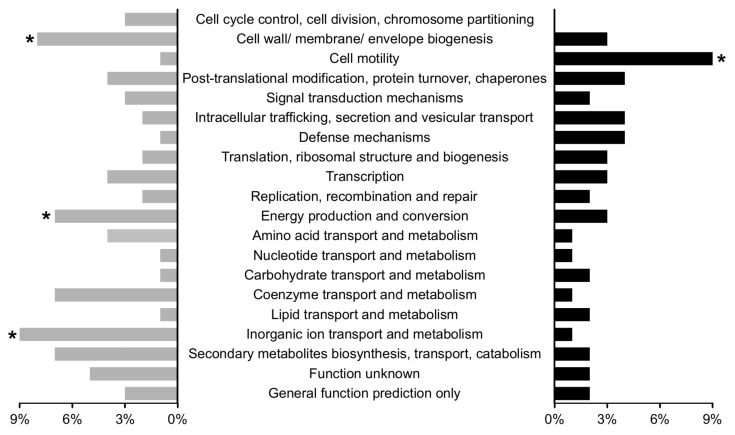
Functional categories of the top 500 differentially expressed genes in *P. phymatum* nodules infected by the wild type versus an *rpoN* mutant during symbiosis, with *P. vulgaris* (genes up-regulated in the *rpoN* mt are in black, those that were down-regulated are in grey) according to classification by eggNOG [40]. Percentages were calculated by dividing the number of significantly up-regulated (322) or down-regulated (178) genes in each category by the total number of retained genes in the same category. The asterisks (*) indicate statistical significance (Fischer test, *p*-value < 0.01).

To further dissect the role of RpoN during symbiosis, and to complement the metabolomics data with an additional data type, we investigated its regulon by differential transcript profiling analysis. Therefore, the *P. vulgaris* root nodules infected with a *P. phymatum* wild-type or an *rpoN* mutant strain were processed at 21 dpi and analyzed by transcriptomics. Two independent biological replicates per strain were processed and sequenced. Subsequently, the unique reads mapping to the *P. phymatum* genome were used for a differential gene expression analysis using the *DESeq* package [39]. The analysis revealed that among the top 500 differentially expressed genes (*DESeq* analysis *p*-value ≤ 0.02, with log_2_ (FC) ≥ 1.35 and ≤ −1.25), 322 genes were positively regulated by RpoN. Of these regulated *P. phymatum* genes, 39% were located on chromosome 1 (3.48 Mb), 22% on chromosome 2 (2.7 Mb), 14% on plasmid 1 (1.9 Mb), and 25% on the symbiotic plasmid (0.59 Mb) (Figure S3). This indicated a significant enrichment of positively-regulated genes on the symbiotic plasmid, which only accounts for 6% of all *P. phymatum* genes (Figure S3). To categorize these differences into modules of biological relevance, the top 500 differentially regulated genes were assigned to functional categories (Figure 2).

Among the top 500 regulated genes, 178 were up-regulated in nodules induced by the *rpoN* mutant. The eggNOG [40] category “cell motility” was the only category found to be significantly over-represented among these genes (Figure 2); the category includes flagella structural genes (Bphy_2931, *fliF*; Bphy_2937, *fliL*; Bphy_2949-51, *fliCD*) and a regulatory gene (Bphy_2962, *flhF*). It is known that most bacteria are able to directionally move in response to different external stimuli, e.g., to swim away from harmful compounds [41]. The up-regulation of flagellar genes in this case might be due to the hostile environment in which the Fix^−^ bacteroids are embedded. Indeed, metabolomics analysis revealed an accumulation of flavonoids in nodules induced by the *rpoN* mutant strain, which were shown to be potentially noxious for other bacteria [42,43]. In line with the suggestion that *rpoN* mutant bacteroids undergo stressful conditions, several resistance-nodulation-division (RND) efflux transporter genes (Bphy_1582, Bphy_3489, Bphy_3490, Bphy_3492, Bphy_4022, and Bphy_4998) [44], as well as several genes involved in the response to oxidative stress (the alkyl hydroperoxide reductases Bphy_1001 and Bphy_3656) were up-regulated. In contrast, among the 322 genes positively controlled by RpoN, the three categories “cell wall/membrane/envelope biogenesis”, “energy production and conversion”, and “inorganic ion transport and metabolism” were over-represented (Figure 2). Within the category “cell wall/membrane/envelope biogenesis”, a cluster involved in the formation of peptidoglycan (PG) (Bphy_2672-78) and the d-alanine:d-alanine ligase encoding gene Bphy_2671 (*ddl*) were identified. Moreover, two potential gene clusters (Bphy_1676-95 and Bphy_2458-76) coding for exopolysaccharides (EPS) and lipopolysaccharides (LPS), respectively, as well as genes coding for several glycosyltransferases (Bphy_1681, Bphy_2460, Bphy_2464, Bphy_2468-69, Bphy_3557, and Bphy_7707) were found. The two categories “energy production and conversion” and “ion transport and metabolism” had already previously been shown to be over-represented during symbiosis with *P. vulgaris*, when free-living cells were used as baseline [23]. In fact, the functional category “energy production and conversion” includes genes with important symbiotic functions, such as nitrogen fixation (*nif* cluster), hydrogenase (*hyd* cluster), and a cytochrome o ubiquinol oxidase operon (*cyoABCD*). Other genes encoding an ATP synthase (Bphy_3029, Bphy_3031-32) and a pyruvate dehydrogenase (Bphy_3758-60) were also found in the same category, and showed induced and RpoN-dependent expression during symbiosis. Among RpoN-activated genes, the over-represented category “inorganic ion transport and metabolism” included *amtB* (Bphy_0257) encoding an ammonium transporter, genes coding for different ATP-binding cassette (ABC)-transporters for sulfate (Bphy_1627, Bphy_1629 and Bphy_7234-36), nitrate/sulphonate/bicarbonate (Bphy_3603), aliphatic sulphonate (Bphy_5227 and Bphy_5229), taurine (Bphy_6080), and urea (Bphy_2251-52 and Bphy_2255). Moreover, several regulatory genes were found among the top 500 genes showing reduced expression in Fix^−^ nodules (Table S3): *ntrB* and *ntrC*, which are part of a two-component regulatory system (2CRS), known to be relevant for nitrogen metabolism in several bacteria (s. below) [45]; Bphy_1669 coding for a cyclic AMP receptor protein (Crp)-fumarate and nitrate reduction regulator (FNR) family transcriptional regulator; six LysR-type transcriptional regulators; and seven regulators of the AraC family.

A large overlap of 52% (168 genes) was identified among the 322 genes positively regulated by RpoN during symbiosis and the genes with increased expression during symbiosis with *P. vulgaris*, compared to free-living conditions reported earlier [23] (Figure S4 and Table S4). On top of the *nif*, *hyd*, and *cyo* genes, several genes from the functional category “inorganic ion transport” described above were found to be co-regulated. Among them, we found several ATP-binding cassette (ABC)-transporter genes for sulfate (Bphy_1627 and Bphy_1629), nitrate/sulphonate/bicarbonate (Bphy_3603), aliphatic sulphonate (Bphy_5227 and Bphy_5229), taurine (Bphy_6080), and urea (Bphy_2251-52). Furthermore, the expression of two transcriptional regulatory genes, *ntrC* and Bphy_1669, was induced in nodules (compared to roots) and RpoN-dependent.

Q-PCR analysis was performed on selected genes showing increased, decreased, and unchanged expression in Fix^−^ nodules compared to Fix^+^. In all cases, the trend of the RNA-Seq results could be confirmed (Table S5).

In summary, the symbiotic RpoN regulon of *P. phymatum* showed a prominent overlap with RpoN regulons, determined previously using microarrays in nodules formed by two α-rhizobial strains (*Bradyrhizobium diazoefficiens* and *Rhizobium etli*) [11,29,46]. Most notable were the activation of nitrogenase and hydrogenase gene expression, the activation of genes involved in respiration, and the transport of ammonium.

### 2.3. In Silico Identification of RpoN Binding Sites

**Figure 3:**
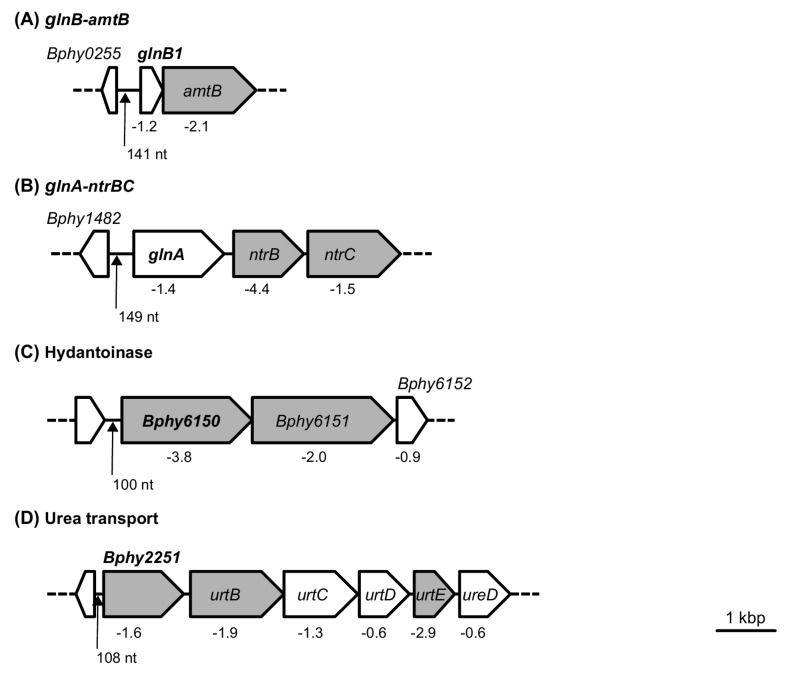
Selected *P. phymatum* gene clusters harboring a putative but high-scoring RpoN binding box in their promoter region. The operon containing the ammonium transporter gene *amtB* (**A**), the operon containing the 2CRS NtrBC (**B**), the genes coding for a hydantoinase (**C**), and the cluster for urea transport (**D**) are shown. Gene names are indicated in italics, while the genes containing an RpoN binding box upstream are shown in bold. Genes present among the top 500 regulated genes (Table S3) are colored in grey, and their log_2_ fold expression changes are shown below. Black arrows indicate the position of the RpoN box; the distance (in nucleotides) from the middle of the box to the translation start site is indicated below the arrow.

A genome-wide in-silico search for RpoN DNA binding motifs (**GG**CACG-N4-TT**GC**) in all *P. phymatum* promoter sequences was conducted, relying on a previously described position-specific frequency matrix (PSFM) of RpoN binding sites in the closely related strain *B. cenocepacia* H111 [27]. A total of 409 putative RpoN binding boxes were detected (see Section 3). Interestingly, 86 out of the 322 genes down-regulated in nodules infected by the *rpoN* mutant showed a potential RpoN binding site in their promoter region, and might therefore be directly controlled by this sigma factor. Several of the genes that showed RpoN-dependent expression in root nodules also contained a highly significant (*p*-value ≤ 6.58 × 10^−5^) RpoN binding sequence in their promoter region: the promoter of Bphy_0256, coding for nitrogen regulatory protein P-II (*glnB1*), which is localized upstream of the ammonium transporter gene (*amtB*) (Figure 3A, Table 3); the promoter of the glutamine synthetase encoding gene (Bphy_1481, *glnA*); followed by two genes of the 2CRS NtrBC (Figure 3B), which were also present among the 322 top down-regulated genes. Moreover, a highly significant RpoN-binding sequence was localized in front of Bphy_2251, which encodes an urea ABC transporter (Figure 3D), and upstream of an operon coding for a hydantoinase (Bphy_6150-52, Figure 3C), which usually hydrolyzes cyclic amide bonds [47]. In a previous study on *B. diazoefficiens* symbiotic gene expression, a gene coding for a hydantoin utilization protein was also found to be a direct RpoN target [11]. Other genes harboring a putative RpoN binding motif in the promoter region that were down-regulated in Fix^−^ nodules are listed in Table 3. These included a gene in the hydrogenase cluster (Bphy_7265, *hydA*) and genes in the *nif* cluster (Bphy_7729, *nifE*; Bphy_7741, *nifV*; Bphy_7742, *nifB*; Bphy_7753, *nifH_1_* and Bphy_7808, *nifH_2_*). The potential RpoN binding motif in *P. phymatum* is illustrated in Figure S5.

### 2.4. Integration of Metabolomics and Transcriptome Data

Several changes in *P. phymatum* transcript expression were supported by respective metabolic alterations. For example, the up-regulation of the citrate synthase gene *gltA* (Bphy_5205) was in agreement with the accumulation of aconitate and isocitrate in nodules induced by the *rpoN* mutant. The decrease in oxo-glutarate, and the increase in glyoxylate in nodules containing the *rpoN* mutant, suggested that the glyoxylate cycle is more active in nodules induced by this Fix^−^ mutant. Indeed, the isocitrate lyase encoding gene (Bphy_1368), which catalyzes the cleavage of isocitrate to succinate and glyoxylate, showed increased expression in nodules infected by the Fix^−^ mutant.

The down-regulation of the d-alanine:d-alanine ligase encoding gene *ddl* (Bphy_2671) and of the downstream *mur* cluster coding for the cell wall component murein or peptidoglycan (PG) was in line with a decrease in the level of the metabolite d-alanyl:d-alanine in nodules induced by the *rpoN* mutant. The d-alanine:d-alanine ligase catalyzes the dimerization of two d-alanine molecules to generate the dipeptide d-alanyl:d-alanine, which is involved in the biosynthesis of PG. The level of this dipeptide was also increased in nodules compared to roots, and, accordingly, we have previously shown that Bphy_2671 had increased expression in root nodules compared to free-living conditions [23]. Interestingly, the results from Wu and colleagues, who showed that certain flavonoids, such as apigenin—which is accumulating in Fix^−^ nodules—target and inhibit the activity of the d-alanine:d-alanine ligase by competing with ATP [48], support our observations. We can therefore speculate that the accumulation of flavonoids in nodules induced by the *rpoN* mutant may lead to defective PG biosynthesis in Fix^−^ bacteroids.

The reduced amount of pentose in Fix^−^ nodules is in line with an increased expression of the ribose-phosphate pyrophosphokinase encoding gene Bphy_0315, as well as with an increased level of several intermediates in the purine metabolism (e.g. inosine and ITP). The degradation of purines leads to the formation of ureides, which are key nitrogen compounds for the growth and yield of legumes forming determinate nodules [49]. The decrease in the level of the ureide allantoic acid in Fix^−^ nodules is supported by an up-regulation of Bphy_2595 coding for an allantoicase, and by an increased level of glyoxylate. Allantoin and allantoic acid were both among the compounds accumulating in *P. phymatum*–*P. vulgaris* nodules compared to uninfected roots (Table S1).

In agreement with reduced amounts of the valine precursor ketovaline in Fix^−^ nodules, we found an increased expression of the 2-isopropylmalate synthase gene, whose product converts ketovaline into isopropylmalate (*leuA*, Bphy_2017). Interestingly, valine was one of the nitrogen sources that was better used by *P. phymatum* under micro-oxic growth conditions (Table S2).

In nodules induced by the *rpoN* mutant, both the level of lipoamide and the expression of the Bphy_3757-61 cluster coding for a pyruvate dehydrogenase significantly decreased. Moreover, the search for RpoN binding boxes detected a potential binding site in the promoter region of Bphy_3761. In *B. diazoefficiens*–soybean root nodules, the expression of the pyruvate dehydrogenase complex was also RpoN-dependent, and the promoter displayed a clear RpoN binding site [11], suggesting direct control of RpoN on this key enzyme in both α- and β-rhizobia.

The reduced amounts of several fatty acyl-CoA molecules in Fix- nodules is in agreement with an upregulation of the enoyl-CoA hydratase encoding gene Bphy_4071, and of the adjacent Bphy_4072 coding for a protein with a acyl-CoA dehydrogenase domain. Both enzymes are important to metabolizing fatty acids to generate both acetyl CoA and energy.

The level of glutamine, which was increased in nodules compared to roots, depends on a functional RpoN. In fact, in Fix^−^ nodules glutamine was detected in lower amounts, and the expression of two glutamine synthetase encoding genes—Bphy_1481 (*glnA*, Figure 3B) and Bphy_7784—also decreased. Accordingly, an increase in the level of glutamate was observed in Fix^−^ nodules. This last result strongly suggested that RpoN is controlling the conversion of glutamate into glutamine in *P. vulgaris* root nodules.

### 2.5. Construction and Phenotypical Analysis of an ntrB Mutant

#### 2.5.1. Role of *ntrB* during Symbiosis

The gene *ntrB* is part of the 2CRS NtrBC, and codes for the sensor kinase necessary for the control of nitrogen metabolism in several bacteria [50,51]. Its companion, transcriptional regulator NtrC, is located downstream, and usually acts together with the sigma factor RpoN to regulate the transcription of several genes. As mentioned above, the expression of *ntrB* and *ntrC* was significantly down-regulated in nodules infected by the *rpoN* mutant versus *P. phymatum* wild-type nodules. Furthermore, *ntrB* had previously been shown to be up-regulated during symbiosis with bean compared to free-living conditions [23]. The presence of a putative RpoN binding box in the promoter region (Figure 3B) led us to investigate the role of NtrB during symbiosis, by constructing a mutant strain.

**Figure 4:**
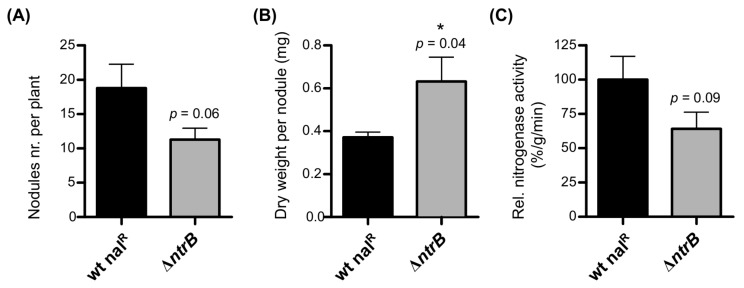
Comparison of the symbiotic properties of *P. vulgaris* plants inoculated with a *ntrB* deletion mutant (Δ*ntrB*) with those of the *P. phymatum* nalidixic acid-resistant wild-type (wt nal^R^) strain. Number of nodules per plant (**A**), dry weight per nodule (**B**), and relative nitrogenase activity (**C**) were determined at 21 days post-infection (dpi). Here, the combined results of two independent experiments are shown. Error bars indicate the standard error of the mean (SEM). The two columns were analyzed by an unpaired student *t*-test (*p*-values are indicated above the SEM, * indicates *p*-value < 0.05).

The *ntrB* deletion mutant (Δ*ntrB*), as well as the nalidixic acid-resistant wild type (wt nal^R^), were inoculated on seedlings of *P. vulgaris*. After three weeks of growth in a plant chamber, several symbiotic parameters were determined, including the number of nodules per plant, the dry weight per nodule, and the relative nitrogenase activity (Figure 4). A lower number of nodules, but with a significantly higher dry weight per nodule, were counted on plants infected by Δ*ntrB*, compared to the ones infected by the nalidixic acid-resistant wild type (Figure 4A,B). The nodules induced by the Δ*ntrB* mutant showed a 36% reduction in the relative nitrogenase activity compared to wild-type nodules (Figure 4C). However, this reduction was not statistically significant (when choosing a significance level α of 0.05). These results suggested that, contrary to RpoN, NtrB from the NtrBC 2CRS plays a marginal role for an efficient establishment of a symbiosis with bean.

#### 2.5.2. Role of *ntrB* for Nitrogen Uptake in Free-Living Conditions

**Figure 5:**
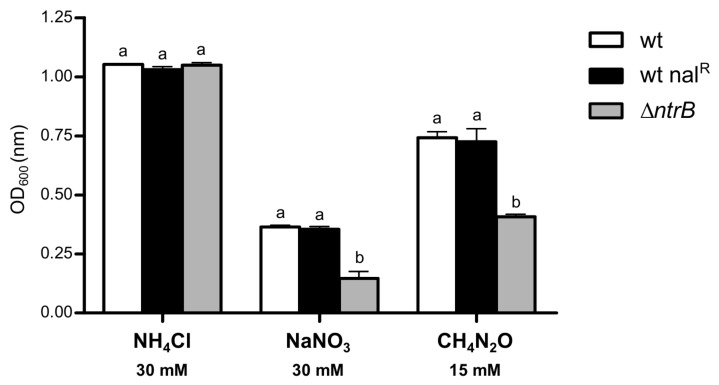
Utilization of selected nitrogen sources by *P. phymatum* wild-type (wt), nalidixic acid-resistant wild type (wt nal^R^), and by a *ntrB* deletion mutant strain (Δ*ntrB*). Growth was assessed with at least two independent replicates. Error bars indicate standard deviation (SD). For each group of columns, values with the same letter are not statistically different, while those with different letters are (ANOVA, Tukey’s test, *p* < 0.001).

To further investigate the role of *P. phymatum ntrB*, the ability to assimilate different nitrogen sources—such as ammonium, nitrate, and urea—was examined in free-living conditions. We found that the growth of the wild type was virtually undistinguishable to that of the nalidixic acid-resistant wild type for all of the three tested nitrogen sources (Figure 5). In contrast, the Δ*ntrB* mutant had a growth defect in the presence of nitrate and urea, but not with ammonium as a nitrogen source (Figure 5). Therefore, in line with previous studies in other bacteria [26,52], *P. phymatum* NtrB—similar to RpoN—is involved in the assimilation of nitrate and urea.

## 3. Materials and Methods

### 3.1. Bacterial Strains, Media, and Cultivation

The bacterial strains, plasmids, and primers used in this work are listed in Table S6. *Escherichia coli* cells were cultivated in a modified Luria–Bertani liquid medium (LB [53]; 10 g of tryptone, 5 g of yeast extract, and 4 g NaCl per liter), whereas *P. phymatum* was grown aerobically in the LB medium without salt. The antibiotics chloramphenicol (20 µg/mL for *E. coli* and 80 µg/mL for *P. phymatum*), kanamycin (25 µg/mL for *E. coli* and 50 µg/mL for *P. phymatum*), and nalidixic acid (50 µg/mL for *P. phymatum*) were used.

The growth of two *P. phymatum* strains—a wild type spontaneously resistant to nalidixic acid (wt nal^R^) and a deletion mutant in *ntrB* (Δ*ntrB*)—were tested in a defined buffered AB minimal medium [54], with 10 mM of sodium citrate as carbon source, and further supplemented with one of the following nitrogen sources, respectively: 30 mM ammonium (Sigma-Aldrich, St. Louis, MO, USA), 30 mM nitrate (Sigma-Aldrich), and 15 mM urea (Sigma-Aldrich). For each strain, the growth from at least two independent cultures was measured. The spectrophotometer Ultrospec 2100 pro (Amersham Biosciences, Little Chalfont, UK) was used.

### 3.2. Plant Growth Conditions

Seeds of the common bean (*Phaseolus vulgaris*, cv. Negro jamapa) were surface-sterilized, as previously described [55]. Germination, inoculation, and the growth conditions for the legumes were conducted as reported previously [23]. Legumes were inoculated with wild-type *P. phymatum*, the nalidixic acid-resistant wild type (wt nal^R^), the *rpoN* mutant [23], and the *ntrB* deletion strain (Δ*ntrB*).

### 3.3. Plant Harvesting and Metabolite Extraction

For comparison of metabolite abundances, bean root nodules induced by different strains (*P. phymatum* wild-type and *rpoN* mutant) and uninfected roots were collected at 21 dpi. Three biological replicates, each in two technical replicates, were analyzed. Around 30 mg nodules or roots were used per sample. Nodules and uninfected roots were flash-frozen in liquid nitrogen immediately after harvesting. Samples were further processed to extract hydrophilic metabolites (methanol extract) as previously described [16].

### 3.4. Metabolite Data Analysis

The methanol extracts were injected in an Agilent 6550 QTOF instrument (Agilent Technologies, Santa Clara, CA, USA) and analyzed by non-targeted flow injection–time-of-flight mass spectrometry in negative mode ionization, as described [33]. Ions were annotated as previously described [16]. Given the existence of metabolites with identical molecular formula and weight, a larger number of candidate metabolites compared to ions was expected. In fact, a total of 409 ions with distinct *m*/*z* values were matched to 493 deprotonated molecules, which were then subjected to statistical analysis. Table S7 contains an overview of all metabolomics raw data, including ions, annotations, and intensities. A comparative statistical analysis was performed, according to Storey and colleagues [56], applying a two-tailed and heteroscedastic *t*-test, followed by the application of a false discovery rate (FDR) correction. A metabolite was considered to be significantly differentially abundant if the following requirements were satisfied: abs[log_2_(fold-change)] ≥ 0.5 and *q*-value < 0.01. Pathway enrichments were calculated using the procedure described previously, using the plant and bacterial KEGG databases [57].

### 3.5. RNA-Sequencing and Data Processing

A modified hot acid phenol protocol [11] was used to extract the total RNA from flash-frozen root nodules induced by the wild type and the *rpoN* mutant (approximately 40 nodules per sample). An additional acid phenol treatment was performed for each sample. Two independent biological replicates were analyzed per condition. Next, complete removal of genomic DNA (gDNA) by DNase treatment and a quality check of the total RNA were performed [27]. The samples were subjected to plant ribosomal RNA (rRNA) removal with the Ribo-zero^TM^ Plant-Seed/Root kit (Epicentre, Madison, DC, USA [23], prior to cDNA synthesis, with a total of 150 ng of high-quality RNA. As previously reported, the library preparation and purification was performed with the Encore Complete Prokaryotic RNA-Seq DR Muliplex System (NuGEN, San Carlos, CA, USA) [23]. The cDNA libraries were quantified using a TapeStation (Agilent Technologies) and sequenced with a HiSeq2500 instrument, single end and 125 base-pair (Illumina, San Diego, CA, USA) [23]. The sequencing reads were trimmed to 70 bp, and further processed and mapped to the *P. phymatum* STM815^T^ genome [5], utilizing the CLC Genomics Workbench v7.0 (QIAGEN CLC bio, Aarhus, Denmark), allowing up to two mismatches per read. The *DESeq* R-package (version 1.30.0) [39] was employed to statistically analyze the reads, uniquely mapping to the genome for differential expression [39]. From the *DESeq* analysis, the top 500 significantly RpoN regulated genes (ranked by ascending *p*-value) were selected. Table S8 contains a complete list of all *P. phymatum* genes and the log_2_ of the fold changes (FC), comparing expression in nodules induced by an *rpoN* mutant versus expression in wild-type nodules. EggNOG v3.0 was used to group the differentially-expressed genes into functional categories [40].The RNA-seq raw data files of nodules induced by both the wild type and by the *rpoN* mutant are accessible through the GEO Series, accession number GSE111993.

### 3.6. q-PCR Analysis

The differential gene expression of the following *P. phymatum* genes—Bphy_0257 (*amtB*), Bphy_1479 (*ntrC*), Bphy_7808 (*nifH_2_*), Bphy_3941 (*rpoD*) and Bphy_3492—was tested by quantitative reverse transcription-PCR (qRT-PCR) [27]. cDNAs were synthesized from an independent biological replicate, as described before [58]. Then qPCR was performed using Brilliant III Ultra-Fast SYBR green QPCR master mix (Agilent Technologies) and a Mx3000P instrument (Agilent Technologies,). For each cDNA sample, three dilutions—15, 7.5, and 3.75 ng/µL—were employed as templates and tested in triplicate. The sigma factor *rpoD* was used to normalize the fold changes in expression, which were calculated with the ΔΔ*C*_t_ method [59]. An annealing temperature of 58 °C was set for all the primers pairs tested.

### 3.7. Genome-Wide In Silico Prediction of RpoN-Binding Sequences

The R-package TFBSTools (version 1.14.2) [60] was employed to predict RpoN binding sites, within a window of 300 nucleotides (nts) upstream of the translation start site of each gene/operon in the *P. phymatum* genome. For the analysis, a minimum score of 85% was set as cut-off. The position-specific frequency matrix (PSFM) reported for RpoN of the closely related *Burkholderia cenocepacia* H111 [27] was used as input.

### 3.8. Construction of a Paraburkholderia Phymatum STM815^T^ ntrB Deletion Mutant

The DNeasy Blood and Tissue kit (Qiagen, Hilden, Germany) was utilized for the extraction of gDNA from *P. phymatum.* To construct a *ntrB* deletion mutant, two fragments flanking the gene *ntrB* were chosen as recombination sites for deletion. The upstream fragment 1 (666 nt) was amplified with primers Bphy1480_1 and Bphy1480_2, and the downstream fragment 2 (692 nt) was amplified with primers Bphy1480_3 and Bphy1480_4, using the gDNA of *P. phymatum* as a template. Both fragments were digested with *Xba*I, ligated, and cloned into the pGEM-T easy vector (Promega, Madison, DC, USA). A kanamycin resistance cassette was cut out from pKD4 [61] plasmid, using *Xba*I, and ligated in between the two fragments on the pGEM-T vector (Promega, Madison, DC, USA). Then, the whole insert was released by digestion with *Kpn*I and sub-cloned into pSHAFT2 [62]. The resulting plasmid was mobilized into a previously-constructed, spontaneous, nalidixic acid-resistant *P. phymatum* strain [23]. The *ntrB* deletion mutant (Δ*ntrB*) was confirmed with PCR, using primers Km_F and glnA_R.

### 3.9. Determination of Symbiotic Properties

Nodule number, nodule dry weight, and nitrogenase activity were calculated as described previously [23,63]. Two independent experiments, with at least nine plants per strain, were performed.

### 3.10. Biolog Analysis

Biolog Phenotypical MicroArray (Biolog, Hayward, CA, USA) for carbon (PM1, 2a) and nitrogen (PM3b) utilization were performed as described [64]. After two days incubation at 30 °C on R-2A agar (Sigma), *P. phymatum* cells were prepared for the inoculation of the Biolog PM plates. The PM plates were then incubated for three days at 30 °C under micro-oxic conditions, using the BD GasPak™ system jar (Becton Dickinson, Franklin Lakes, NJ, USA) by changing the sachet once a day. Afterwards, the OD_590_ of each well was measured using a plate-reader.

## 4. Conclusions

**Figure 6:**
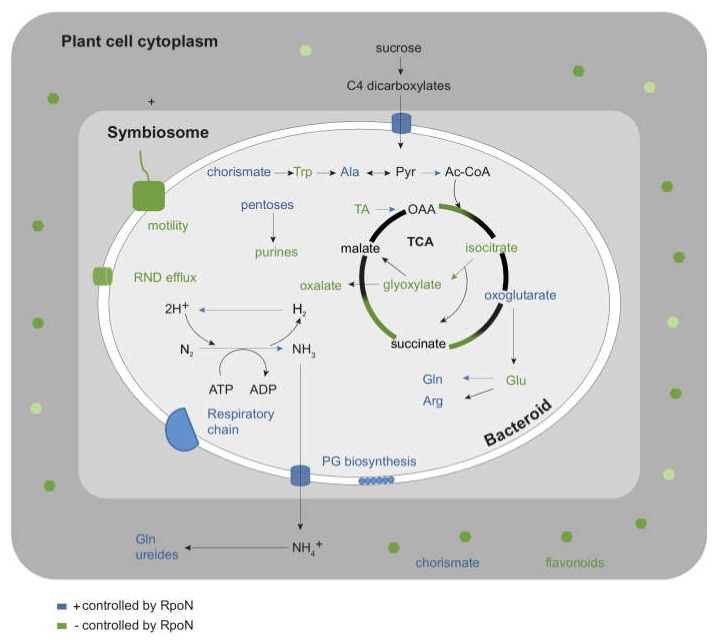
Scheme of the main changes in metabolites and transcripts profile in *P. vulgaris* root nodules infected by a *rpoN* mutant, compared to wild-type nodules. Metabolites and reactions down- and up-regulated in nodules induced by the *rpoN* mutant are indicated in blue and green, respectively. Glu: glutamate; Gln: glutamine; OAA: oxaloacetate; Trp: tryptophan; Ala: alanine; Pyr: pyruvate; Arg: arginine; RND: resistance-nodulation-division; PG: peptidoglycan.

In this study, we aimed to dissect the regulon of *P. phymatum* RpoN, the key sigma factor of nitrogen-fixing symbiosis, by analyzing and integrating metabolite changes with changes in gene expression in *P. vulgaris* root nodules. The importance of RpoN for symbiosis, both at the metabolite and transcript level, was evidenced by a large overlap of nodule-specific metabolites/transcripts with RpoN-dependent metabolites/transcripts (90% and 52%, respectively). Furthermore, we found numerous examples where the different metabolite abundances correlated with *P. phymatum* genes expression differences (e.g., PG biosynthesis, conversion of glutamine in glutamate, glyoxylate cycle) (Figure 6). Interestingly, we found that nodules induced by the Nod^+^ Fix^−^ mutant seemed to accumulate significant amounts of flavonoids, which may be used as a defense reaction by the plant, which probably recognizes this strain as a Fix^−^ cheater strain. This hypothesis is supported by the *rpoN* mutant-induced up-regulation of several *P. phymatum* RND efflux transporter genes, as well as other genes involved in the resistance to stress inside nodules. The effects of accumulating flavonoids on *P. phymatum* growth could be tested by adding different concentrations of flavonoids to free-living cultures. Microscopic analysis to investigate the ultrastructure of Fix^+^ and Fix^−^ nodules may help to gain more information about the status of their bacteroids. This first metabolome analysis of β-rhizobial root nodules contributed new insights on the differences between the physiology of nodules induced by α-and β-rhizobia. Glutamate, for example, was among the most abundant metabolites in nodules of several α-rhizobial symbiosis model systems [16,65,66,67,68], but did not show differential abundance in bean nodules induced by *P. phymatum*.

In order to better characterize the adaptation of *P. phymatum* to papilionoid legumes, metabolomics studies should also be performed on nodules formed by *P. phymatum* on its native mimosoid host plant in the near future.

## Figures and Tables

**Table 1:** Enriched pathways for metabolites differentially accumulating in nodules induced by the *rpoN* mutant (*rpoN* mt nod), versus nodules occupied by the wild type (wt nod) and vice versa. The enrichment analysis was performed using plant and bacterial metabolites databases.

Enriched KEGG Pathway ^1^	*rpoN* mt nod > wt nod	wt nod > *rpoN* mt nod
	*q*-value ^2^	*q*-value ^3^
Plant KEGG database		
Flavonoid biosynthesis	1.44 × 10^−8^	
Citrate cycle (TCA cycle)	1.88 × 10^−4^	
Glyoxylate and dicarboxylate metabolism	1.72 × 10^−3^	
Isoflavonoid biosynthesis	2.13 × 10^−3^	
Ascorbate and aldarate metabolism	3.15 × 10^−3^	
Brassinosteroid biosynthesis	3.15 × 10^−3^	
Ubiquinone and other terpenoid-quinone biosynthesis	9.51 × 10^−3^	
Fatty acid elongation		4.12 × 10^−4^
Lysine degradation		4.89 × 10^−4^
Fatty acid degradation		1.56 × 10^−3^
Lysine biosynthesis		1.81 × 10^−3^
Glycine, serine and threonine metabolism		1.84 × 10^−3^
Butanoate metabolism		1.27 × 10^−2^
Cyanoamino acid metabolism		1.59 × 10^−2^
Glycerophospholipid metabolism		1.59 × 10^−2^
Ether lipid metabolism		1.59 × 10^−2^
Bacteria KEGG database		
Citrate cycle (TCA cycle)	2.10 × 10^−2^	
Fatty acid metabolism		1.96 × 10^−3^
Microbial metabolism in diverse environments		1.12 × 10^−2^
Lysine degradation		1.12 × 10^−2^
Glycine, serine and threonine metabolism		1.19 × 10^−2^
ABC transporters		1.19 × 10^−2^
Lysine biosynthesis		1.19 × 10^−2^
Butanoate metabolism		1.47 × 10^−2^
Caprolactam degradation		1.47 × 10^−2^
Glycerophospholipid metabolism		1.50 × 10^−2^
Valine, leucine and isoleucine biosynthesis		1.61 × 10^−2^
Aminoacyl-tRNA biosynthesis		1.61 × 10^−2^
Aminobenzoate degradation		1.78 × 10^−2^
Biosynthesis of secondary metabolites		1.78 × 10^−2^
Histidine metabolism		1.78 × 10^−2^
Cyanoamino acid metabolism		1.78 × 10^−2^
Arginine and proline metabolism		1.78 × 10^−2^
Sulfur metabolism		1.95 × 10^−2^
Valine, leucine and isoleucine degradation		1.95 × 10^−2^
Vitamin B6 metabolism		2.16 × 10^−2^

^1^ The Kyoto Encyclopedia of Genes and Genomes (KEGG) pathways were taken from http://www.genome.jp/kegg/pathway.html; ^2^ only categories enriched with a *q*-value ≤ 0.02 among metabolites showing a statistically significant increase in nodules induced by the *rpoN* mutant (*rpoN* mt nod) in comparison with nodules induced by the wild type (wt nod) are listed; ^3^ like ^2^, but here for categories of metabolites showing a statistically significant decrease in nodules induced by the *rpoN* mt.

**Table 2:** List of 147 metabolites that significantly differentially accumulated in *P. vulgaris* nodules, induced by a *P. phymatum* wild-type (wt nod) and by an *rpoN* mutant strain (*rpoN* mt nod).

Metabolites ^1^	ID ^1^	log_2_FC (*rpoN* mt nod vs. wt nod) ^2^
More abundant in nodules induced by the *rpoN* mutant		
Naringenin	C00509	3.0
2-*C*-Methyl-d-erythritol 2,4-cyclodiphosphate	C11453	2.4
2-*C*-Methyl-d-erythritol 4-phosphate	C11434	2.3
Homoeriodictyol chalcone	C16405	2.1
1-Nitronaphthalene-5,6-oxide	C14800	2.0
C22:0	C08281	2.0
Parathion	C06604	1.8
2′,7-Dihydroxy-4′,5′-methylenedioxyisoflavone	C16226	1.7
6-Thiourate	C16613	1.5
Luteolin	C01514	1.5
Apigenin	C01477	1.5
Oxalic acid	C00209	1.5
3-Dehydroteasterone	C15792	1.5
*N*-Acetylneuraminate	C00270	1.4
Phenyl acetate	C01454	1.3
3β-Hydroxy-4β-methyl-5α-cholest-7-ene-4α-carboxylate	C04840	1.3
Afzelechin	C09320	1.3
(−)Vestitone	C00786	1.3
Histidine	C00135	1.2
Phospho*enol*pyruvate	C00074	1.2
Cinnamate	C00423	1.2
Thymidine	C00214	1.2
*N*-Acetyl-d-glucosamine	C00140	1.1
Quinate	C00296	1.1
3,9-Dihydroxypterocarpan	C04271	1.1
Furoic acid	C01546	1.1
Aconitate	C00417	1.1
(Iso)Citrate	C00158	1.1
Propanoyl phosphate	C02876	1.0
Phosphoaspartate	C03082	1.0
Formamidopyrimidine nucleoside triphosphate	C05922	1.0
6-Deoxoteasterone	C15799	1.0
*O*-Succinyl-l-homoserine	C01118	1.0
Aspartate	C00049	0.9
UDP-6-sulfoquinovose	C11521	0.9
γ-Tocopherol	C02483	0.9
*sn*-Glycerol 3-phosphate	C00093	0.9
Teasterone	C15791	0.8
ITP	C00081	0.8
3-Methyl-*cis*,*cis*-hexadienedioate	C04112	0.8
2-Oxo-3-hydroxy-4-phosphobutanoate	C06054	0.8
Leukotriene B4	C02165	0.8
Glutamate	C00025	0.8
4-Maleylacetoacetate	C01036	0.8
1-*O*-Sinapoyl-β-d-glucose	C01175	0.7
Gallate	C01424	0.7
2-Dehydropantoate	C00966	0.7
Chlorogenate	C00852	0.7
5-Hydroxyferulic acid methyl ester	C05619	0.7
3-Methoxyapigenin	C05902	0.7
Indole-3-acetate	C00954	0.7
Biotin	C00120	0.7
Naphthalene-1,2-diol	C03012	0.7
3′,5′-cyclic di-GMP	C16463	0.7
Serine	C00065	0.7
5-l-Glutamyltaurine	C05844	0.6
Tartaric acid	C00552	0.6
5-Hydroxyindoleacetate	C05635	0.6
Tryptophan	C00078	0.6
7-Methyluric acid	C16355	0.6
3,4-Dihydroxyphenylethyleneglycol	C05576	0.6
Cathasterone	C15790	0.6
UDP-deoxyhexose	C02199	0.6
22-Hydroxydocosanoate	C19623	0.6
5-Amino-6-(5′-phospho-d-ribitylamino)uracil	C04454	0.6
1-Phospho-α-d-galacturonate	C04037	0.6
Itaconate	C00433	0.6
UTP	C00075	0.6
Inosine	C00294	0.5
Aminobutanoic acid (ABA)	C00334	0.5
AMP	C00020	0.5
Glyoxylic acid	C00048	0.5
(8*Z*,11*Z*,14*Z*)-Icosatrienoic acid	C03242	0.5
GDP	C00035	0.5
Pseudobaptigenin	C10522	0.5
Succinic aldehyde	C00741	0.5
(6*Z*,9*Z*,12*Z*)-Octadecatrienoic acid	C06426	0.5
Less abundant in nodules induced by the *rpoN* mutant		
Oxobutanoic acid	C00109	−0.5
*N*-Acetylmuramate	C02713	−0.5
7,8-Diaminononanoate	C01037	−0.5
(*R*)-3-((*R*)-3-Hydroxybutanoyloxy)butanoate	C04546	−0.5
Orcinol	C02923	−0.6
3-Hydroxy-5-methylhex-4-enoyl-CoA	C16469	−0.6
FMN (ox)	C00061	−0.6
3-*O*-Methylquercetin	C04443	−0.6
Sinapoyl aldehyde	C05610	−0.6
Octanoyl-CoA	C01944	−0.6
Allantoate	C00499	−0.6
Homoserine lactone	C01234	−0.6
C4:0 (Butyric acid)	C00246	−0.6
5-Aminolevulinic acid	C00430	−0.6
Gibberellin A1	C00859	−0.6
2-Hydroxy-2,4-pentadienoic acid	C07091	−0.7
Coproporphyrinogen III	C03263	−0.7
UDP-3-*O*-(3-hydroxytetradecanoyl)-d-glucosamine	C06022	−0.7
ADP-ribose	C00301	−0.7
Tetradecanoyl-CoA	C02593	−0.7
Estrone 3-sulfate	C02538	−0.7
(9*Z*)-Hexadecenoic acid	C08362	−0.7
2-Deoxy-d-ribose 1-phosphate	C00672	−0.8
(*S*)-3-Hydroxytetradecanoyl-CoA	C05260	−0.8
*N*-Acetyl-l-glutamate	C00624	−0.8
Pipecolate	C00408	−0.9
Ala-Ala	C00993	−0.9
Lysine	C00047	−0.9
*S*-(Formylmethyl)glutathione	C14871	−0.9
5-Amino-4-imidazolecarboxyamide	C04051	−1.0
Pentose	C00121	−1.0
5-Hydroxyisourate	C11821	−1.0
5,7,24(28)-Ergostatrienol	C15778	−1.1
Acrolein	C05986	−1.1
Lipoamide	C00248	−1.2
Sphingosine 1-phosphate	C06124	−1.2
5-Hydroxyectoine	C16432	−1.2
3-Carbamoyl-2-phenylpropionaldehyde	C16587	−1.2
Ketovaline	C00141	−1.2
3-Propylmalate	C02504	−1.2
Dihydrothymine	C05715	−1.3
Acetyl-Glu-semialdehyde	C01250	−1.3
Propenoic acid C3:1	C00511	−1.3
Leukotriene A4	C00909	−1.4
d-2-Hydroxyisocaproate	C06103	−1.4
Nicotinate d-ribonucleotide	C01185	−1.4
Threonine	C00188	−1.4
Ectoine	C06231	−1.4
sn-Glycero-3-phosphocholine	C00670	−1.4
(2*R*)-2-Hydroxy-2-methylbutanenitrile	C18796	−1.5
4,4-Dimethyl-5α-cholesta-8,14,24-trien-3β-ol	C11455	−1.5
Coniferyl aldehyde	C02666	−1.6
Histidinol	C00860	−1.6
2-Hydroxycyclohexan-1-one	C01147	−1.7
3-Phosphonooxypyruvate	C03232	−1.7
3-Methyl-2-butenal	C07330	−1.7
Glyphosate	C11638	−1.8
Oxoglutarate	C00026	−1.8
Isopropylmaleate	C02631	−1.9
Aminoadipate	C00956	−2.2
Cyclohexanone	C00414	−2.3
Butynol	C20701	−2.4
Pyridoxamine phosphate	C00647	−2.5
Alanine	C00041	−2.9
Glutamine	C00064	−2.9
10-Formyl-THF	C00234	−3.0
Diaminopimelate	C00666	−3.2
Ornithine	C00077	−3.2
Arginine	C00062	−3.3
Chorismate	C00251	−4.7

^1^ Metabolite name and ID according to the KEGG dataset; ^2^ log_2_ of the metabolite level fold change (FC), comparing nodules induced by the *rpoN* mutant (*rpoN* mt nod) with wild-type (wt nod) nodules; ADP: adenosine diphosphate; AMP: adenosine monophosphate; FMN: flavin mononucleotide; GMP: guanine monophosphate; ITP: inosine triphosphate; THF: tetrahydrofolate; UDP: uridine diphosphate; UTP: uridine triphosphate.

**Table 3:** List of 93 genes positively controlled by RpoN and belonging to an over-represented eggNOG category (Fischer test, *p*-value < 0.01). Genes harboring a putative RpoN-box in their promoter region are shown in bold.

Locus ID ^1^	Description ^1^	Gene Name	log_2_FC (*rpoN* mt nod vs. wt nod) ^2^
Cell wall/membrane/envelope biogenesis			
Bphy_0649	RND efflux system outer membrane lipoprotein		−5.0
Bphy_0919	NLP/P60 protein		−1.6
Bphy_1282	OmpW family protein		−2.6
Bphy_1546	phospholipase C		−2.1
Bphy_1681	group 1 glycosyl transferase		−2.2
Bphy_1689	exopolysaccharide transport protein family		−3.2
Bphy_1690	polysaccharide export protein		−4.3
Bphy_1691	exopolysaccharide biosynthesis polyprenyl glycosylphosphotransferase		−3.4
**Bphy_2283**	**mannose-1-phosphate guanylyltransferase**		−**2.9**
Bphy_2316	dTDP-4-dehydrorhamnose reductase		−2.3
Bphy_2460	group 1 glycosyl transferase		−2.5
Bphy_2464	group 1 glycosyl transferase		−3.1
Bphy_2468	putative glycosyl transferase		−2.1
Bphy_2469	group 1 glycosyl transferase		−3.1
Bphy_2470	NAD-dependent epimerase/dehydratase		−3.2
Bphy_2471	GDP-mannose 4,6-dehydratase		−3.8
Bphy_2472	exopolysaccharide transport protein family		−3.7
Bphy_2473	polysaccharide export protein		−2.7
Bphy_2474	undecaprenyl-phosphate glucose phosphotransferase		−3.8
Bphy_2475	mannose-1-phosphate guanylyltransferase		−3.2
Bphy_2670	polypeptide-transport-associated domain-containing protein		−1.5
Bphy_2671	d-alanine:d-alanine ligase	*ddl*	−3.0
Bphy_2672	UDP-*N*-acetylmuramate-l-alanine ligase	*murC*	−1.8
Bphy_2678	UDP-*N*-acetylmuramoylalanyl-d-glutamate-2	*murE*	−1.6
Bphy_3069	lytic transglycosylase		−1.9
Bphy_3557	glycosyl transferase family protein		−2.3
Bphy_4515	porin		−2.0
Bphy_5347	NAD-dependent epimerase/dehydratase		−4.7
Bphy_7633	d-alanine:d-alanine ligase		−3.7
Bphy_7707	glycosyl transferase family protein		−3.6
Bphy_7819	porin		−2.9
Energy production and conversion			
Bphy_1284	aldehyde dehydrogenase		−1.8
Bphy_1649	alkanesulfonate monooxygenase		−3.2
Bphy_2012	PIG3 family NAD(P)H quinone oxidoreductase		−1.9
Bphy_2272	FAD linked oxidase domain-containing protein		−2.8
Bphy_3029	F0F1 ATP synthase subunit α		−1.5
Bphy_3031	F0F1 ATP synthase subunit B		−1.6
Bphy_3032	F0F1 ATP synthase subunit C		−2.6
Bphy_3646	cytochrome o ubiquinol oxidase subunit IV	*cyoD*	−3.9
Bphy_3647	cytochrome o ubiquinol oxidase, subunit III	*cyoC*	−5.1
Bphy_3648	cytochrome o ubiquinol oxidase, subunit I	*cyoB*	−4.0
Bphy_3649	ubiquinol oxidase, subunit II	*cyoA*	−4.2
**Bphy_3759**	**transketolase central region**		−**2.3**
**Bphy_3760**	**pyruvate dehydrogenase (acetyl-transferring)**		−**2.5**
**Bphy_4125**	**acylphosphatase**		−**2.8**
**Bphy_4520**	**formate dehydrogenase, γ subunit**		−**1.3**
**Bphy_5148**	**AraC family transcriptional regulator**		−**1.8**
Bphy_5156	l-lactate dehydrogenase (cytochrome)		−1.9
Bphy_5235	alkanesulfonate monooxygenase		−3.5
Bphy_5641	glycolate oxidase iron-sulfur subunit	*glcF*	−2.5
**Bphy_6505**	**formylmethanofuran dehydrogenase subunit A**		−**3.5**
Bphy_7231	cytochrome c class I		−2.6
Bphy_7232	xenobiotic (desulfurization)monooxygenase subunit A		−2.9
**Bphy_7263**	**Ni/Fe-hydrogenase, b-type cytochrome subunit**		−**2.8**
**Bphy_7264**	**nickel-dependent hydrogenase large subunit**		−**4.2**
**Bphy_7265**	**hydrogenase (NiFe) small subunit**	***hydA***	−**4.3**
Bphy_7406	aldehyde dehydrogenase		−5.6
**Bphy_7729**	**nitrogenase MoFe cofactor biosynthesis protein**	***nifE***	−**4.6**
**Bphy_7730**	**nitrogenase molybdenum-cofactor biosynthesis protein**	***nifN***	−**2.9**
**Bphy_7733**	**ferredoxin III, nif-specific**		−**5.0**
**Bphy_7737**	**electron-transferring-flavoprotein dehydrogenase**		−**4.2**
**Bphy_7738**	**electron transfer flavoprotein α/β-subunit**		−**4.5**
**Bphy_7739**	**electron transfer flavoprotein α/β-subunit**		−**5.2**
**Bphy_7754**	**nitrogenase molybdenum-iron protein α chain**	***nifD***	−**4.3**
**Bphy_7755**	**nitrogenase molybdenum-iron protein β chain**	***nifK***	−**4.0**
Bphy_7803	electron transfer flavoprotein α subunit		−3.5
Bphy_7804	electron transfer flavoprotein α/β-subunit		−3.1
Inorganic ion transport and metabolism			
Bphy_0141	CutC family protein		−3.2
**Bphy_0257**	**ammonium transporter**	***amtB***	−**2.1**
Bphy_1627	sulfate ABC transporter inner membrane subunit	*cysW*	−1.7
Bphy_1629	sulfate ABC transporter periplasmic sulfate-binding protein		−1.7
Bphy_1647	ABC transporter-like protein		−2.9
Bphy_1648	transport systems inner membrane component		−2.7
Bphy_2231	sulfate adenylyltransferase large subunit		−2.2
Bphy_2235	sulfite reductase		−2.0
Bphy_3602	ABC transporter related		−1.7
Bphy_3603	ABC transporter periplasmic ligand-binding protein		−2.4
**Bphy_5040**	**NlpA lipoprotein**		−**4.0**
Bphy_5227	ABC-type glycine betaine transport system		−2.8
Bphy_5229	aliphatic sulfonate ABC transporter periplasmic protein		−3.7
Bphy_5232	rhodanese domain-containing protein		−4.0
Bphy_5473	Dyp-type peroxidase family protein		−1.9
Bphy_5555	sulfatase		−1.6
Bphy_6080	taurine ABC transporter, periplasmic binding protein		−4.6
Bphy_7233	ABC transporter related		−3.2
Bphy_7234	transport systems inner membrane component		−3.7
Bphy_7235	transport systems inner membrane component		−3.0
Bphy_7236	ABC sulfate ester transporter, periplasmic protein		−2.5
Bphy_7645	transport systems inner membrane component		−2.6
Bphy_7646	transport systems inner membrane component		−3.1
Bphy_7647	ABC transporter related		−3.1
**Bphy_7753**	**nitrogenase reductase**	***nifH_1_***	−**5.2**
**Bphy_7808**	**nitrogenase reductase**	***nifH_2_***	−**5.2**

^1^ Locus identifier and description was extracted from the GenBank files (NC_010622.1, NC_010623.1, NC_010625.1, NC_010627.1); ^2^ log_2_ of the fold change (FC) in expression of *P. vulgaris* nodules induced by an *rpoN* mutant (*rpoN* mt nod) versus the wild type (wt nod); RND: resistance-nodulation-division; dTDP: deoxythymidine diphosphate; NAD: nicotinamide adenine dinucleotide; GDP: guanosine diphosphate; UDP: uridine diphosphate; FAD: Flavin adenine dinucleotide; ABC: ATP-binding cassette.

## Supplementary Materials

The following materials are available online at http://www.mdpi.com/1422-0067/19/4/1049/s1. Figure S1: Venn diagram showing the overlap between 169 metabolites that are more abundant in nodules infected by *P. phymatum* wild-type (wt) versus bean root, and the 70 metabolites that are less abundant in nodules induced by *P. phymatum rpoN* mutant (*rpoN*) versus the wild-type (wt); Figure S2: Venn diagram showing the overlap between the 62 metabolites that are less abundant in nodules infected by *P. phymatum* wild-type (wt) versus bean root, and the 77 metabolites that are more abundant in nodules induced by *P. phymatum rpoN* mutant (*rpoN*) versus the wild-type (wt); Figure S3: Pie charts representing the percentages of genes distributed in the *P. phymatum* STM815 genome (**A**); In (**B**), the localization of the 322 genes showing down-regulation in nodules induced by the *rpoN* mutant is shown. A statistically significant enrichment of genes located on the symbiotic plasmid is observed; Figure S4: Venn diagram showing the overlap between the 322 genes significantly and positively controlled by RpoN, and the 322 genes up-regulated during symbiosis, compared to free-living conditions (*; Lardi et al., 2017); Figure S5: Consensus sequence logo for *P. phymatum* STM815 RpoN–binding site. Depicted is a putative RpoN–binding site for *P. phymatum*, based on the 409 promoter sequences. The new consensus sequence was created using WebLogo (http://weblogo.berkeley.edu/); Table S1: List of 231 metabolites showing altered abundance in bean root nodules induced by the *P. phymatum* wild-type, compared to the uninfected root; Table S2: C and N source utilization of *P. phymatum* STM815 wild-type during micro-oxic conditions, using Biolog PM plates (1, 2a for C and 3b for N). After three days incubation at 30 °C, optical density at 590 nm was measured for each well; Table S3: List of the top 500 differentially regulated genes in nodules induced by *rpoN* mutant versus wild-type nodules (*DESeq* analysis; *p*-value ≤ 0.02, log2 [FC] ≥ 1.35 and ≤ −1.25); Table S4: List of the 168 genes positively regulated by RpoN during symbiosis and showing induced expression in nodules compared to free-living conditions (Lardi et al., 2017); Table S5: qPCR analysis of several genes. Fold changes in expression of the selected genes in nodules induced by *rpoN* mutant (*rpoN* mt nod) versus expression in wild-type nodules (wt nod) are shown; Table S6: Bacterial strains, plasmids, and oligonucleotides used in this study; Table S7: Raw metabolomics data, including injections, ions, annotations, and intensities; Table S8: List of all *P. phymatum* genes, log_2_ of the fold changes in expression, and the *p*-values in bean nodules infected by *rpoN* mutant versus wild type as assessed by *DESeq* analysis.
