# Supplementary material for: Metabolomics and Transcriptomics Identify Multiple Downstream Targets of Paraburkholderia phymatum σ54 During Symbiosis with Phaseolus vulgaris
Source: Int J Mol Sci. 2018 Apr 1;19(4):1049. doi: 10.3390/ijms19041049 (PMC5979394; doi:10.3390/ijms19041049)
Supplement: Supplementary file 1 [file ijms-19-01049-s001.zip › Supplementary files_images+tables/Table S6_20180328.docx]

# **Table S6:** Bacterial strains, plasmids and oligonucleotides used in this study.

| Strain or plasmid | Description | Reference |
| --- | --- | --- |
| Strains | | |
| *E. coli* |  |  |
| cc118λ-pir | Δ(ara-leu) *araD* Δ*lacX74 galE galK phoA20 thi1 rpsE rpoB argE(Am) recAl* λ pir; Strep^R^ | [1] |
|  |  |  |
| *P. phymatum* |  |  |
| STM815 | Wild type | [2] |
| STM815_NAL_ | Wild type spontaneously resistant to nalidixic acid; Nal^R^ | [3] |
| STM815-rpoN_Pp_ | *rpoN*_Pp_::pSHAFT2 mutant of STM815; Cm^R^ | [3] |
| STM815-ΔntrB_Pp_ | STM815 spontaneously resistant to nalidixic acid with deletion in *ntrB* gene (Bphy_1480); Nal^R^, Cm^R^ | This study |
|  |  |  |
| Plasmids | | |
| pGEM-T Easy | Cloning vector; Amp^R^, *lac*Z | Promega |
| pKD4 | Vector containing kanamycin cassette; Km^R^ | [4] |
| pRK2013 | Helper plasmid; Km^R^ | [5] |
| pSHAFT2 | Suicide plasmid; Cm^R^ | [6] |
| pBBR1MCS-2 | Broad host-range cloning vector; Km^R^ | [7] |
| pSHAFT-*rpoN* | pSHAFT2 containing a 489 bp internal fragment of Bphy_0326 for mutagenesis; Cm^R^ | [3] |
| Δ*ntrB*-pSHAFT | pSHAFT2 containing two fragments of the external region of *ntrB* and a kanamycin cassette; Cm^R^ , Km^R^ | This study |
|  |  |  |
| Oligonucleotides | **Sequence^1^** | **Source** |
| Deletion mutant construction | | |
| pSHAFTseqFor | CTTCAGCTGATGTGTGATAACATACT | K. Agnoli unpublished |
| Bphy1480_1 | TTTTGGTACCTCGGATTCCAGCAGATCTT | This study |
| Bphy1480_2 | TTTTTCTAGAGTCTGACAAACGAGTGCAC | This study |
| Bphy1480_3 | TTTTTCTAGACAATACCATCTTGCAGTC | This study |
| Bphy1480_4 | TTTTGATATCGAATTCGCGCTGTTCTACA | This study |
| glnA_R | TGGACGCAGCAACTGAAGTA | This study |
| Km_F | CTATGAAAGGTTGGGCTTCG | This study |
|  |  |  |
| qPCR | | |
| Bphy3941_F | AGATCGTCAGCGAGAACCAT | [3] |
| Bphy3941_R | TCTGACGGTTGGTTTCCTTC | [3] |
| Bphy7808_F | GGCGTGGACTATGTGTCGTA | [3] |
| Bphy7808_R | GATGCCCTTCGAGATGTTGT | [3] |
| Bphy0257_F | GCTCGTCACAGTGATCTGGA | [3] |
| Bphy0257_R | ACTTCATCCGGTCAGCAAAC | [3] |
| Bphy1479_F | ATCTGCTGGAATCCGAACTG | [3] |
| Bphy1479_R | GCGATTCGAGATTCTGATGTG | [3] |
| Bphy3492_F | GGCGAAAGTCACGTTGATTC | This study |
| Bphy3492_R | GTTCACGCCCTCTTCAATCT | This study |

^1^Underlined nucleotides indicate a restriction site.

**References**

1. Herrero, M.; de Lorenzo, V.; Timmis, K. N. Transposon vectors containing non-antibiotic resistance selection markers for cloning and stable chromosomal insertion of foreign genes in gram-negative bacteria. *J. Bacteriol.* **1990**, *172*, 6557–6567.

2. Moulin, L.; Munive, A.; Dreyfus, B.; Boivin-Masson, C. Nodulation of legumes by members of the β-subclass of Proteobacteria. *Nature* **2001**, *411*, 948–950, doi:10.1038/35082070.

3. Lardi, M.; Liu, Y.; Purtschert, G.; Bolzan de Campos, S.; Pessi, G. Transcriptome analysis of *Paraburkholderia phymatum* under nitrogen starvation and during symbiosis with *Phaseolus vulgaris*. *Genes* **2017**, *8*, doi:10.3390/genes8120389.

4. Datsenko, K. A.; Wanner, B. L. One-step inactivation of chromosomal genes in *Escherichia coli* K-12 using PCR products. *Proc. Natl. Acad. Sci. U. S. A.* **2000**, *97*, 6640–6645, doi:10.1073/pnas.120163297.

5. Figurski, D. H.; Helinski, D. R. Replication of an origin-containing derivative of plasmid RK2 dependent on a plasmid function provided in *trans*. *Proc. Natl. Acad. Sci. U. S. A.* **1979**, *76*, 1648–1652.

6. Shastri, S.; Spiewak, H. L.; Sofoluwe, A.; Eidsvaag, V. A.; Asghar, A. H.; Pereira, T.; Bull, E. H.; Butt, A. T.; Thomas, M. S. An efficient system for the generation of marked genetic mutants in members of the genus *Burkholderia*. *Plasmid* **2017**, *89*, 49–56, doi:10.1016/j.plasmid.2016.11.002.

7. Kovach, M. E.; Elzer, P. H.; Steven Hill, D.; Robertson, G. T.; Farris, M. A.; Roop, R. M.; Peterson, K. M. Four new derivatives of the broad-host-range cloning vector pBBR1MCS, carrying different antibiotic-resistance cassettes. *Gene* **1995**, *166*, 175–176, doi:10.1016/0378-1119(95)00584-1.
