# Supplementary figures and images for: Metabolomics and Transcriptomics Identify Multiple Downstream Targets of Paraburkholderia phymatum σ54 During Symbiosis with Phaseolus vulgaris
Source: Int J Mol Sci. 2018 Apr 1;19(4):1049. doi: 10.3390/ijms19041049 (PMC5979394; doi:10.3390/ijms19041049)

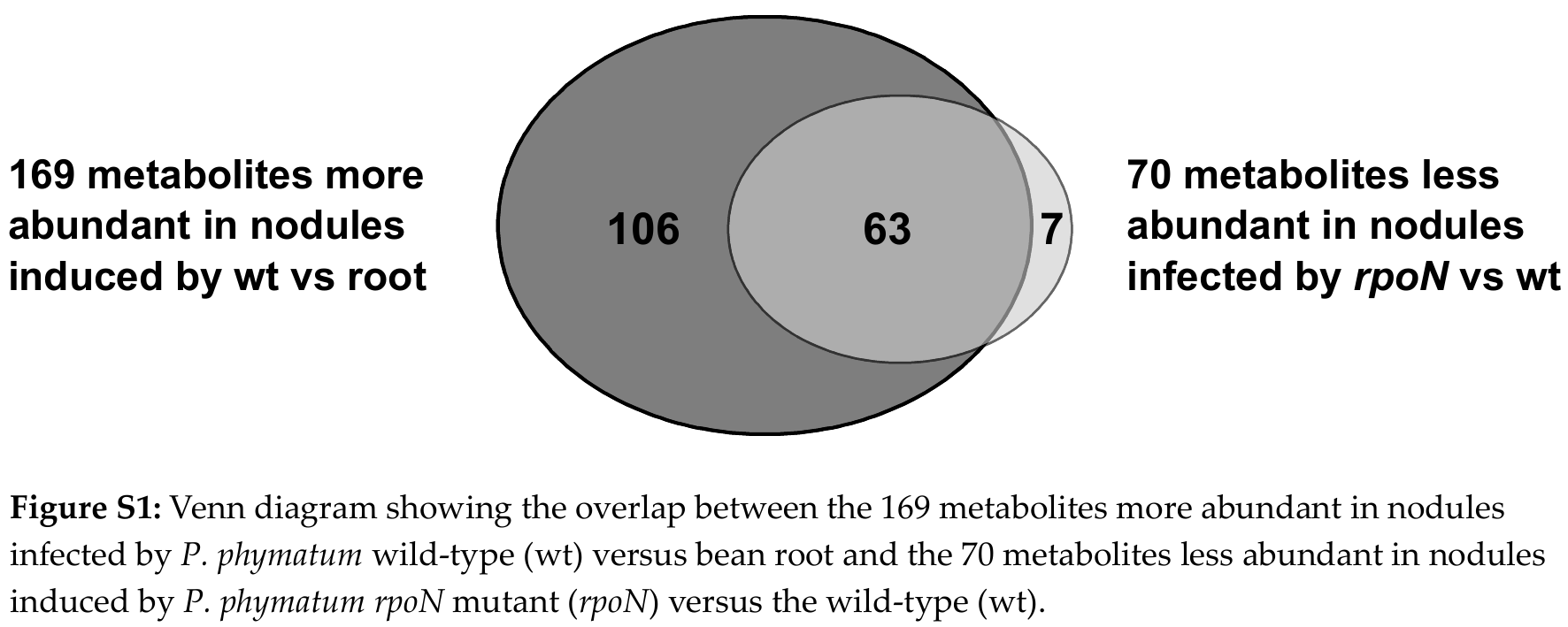

Supplement: Supplementary file 1 [file ijms-19-01049-s001.zip › Supplementary files_images+tables/Figure S1_20180326.tiff]

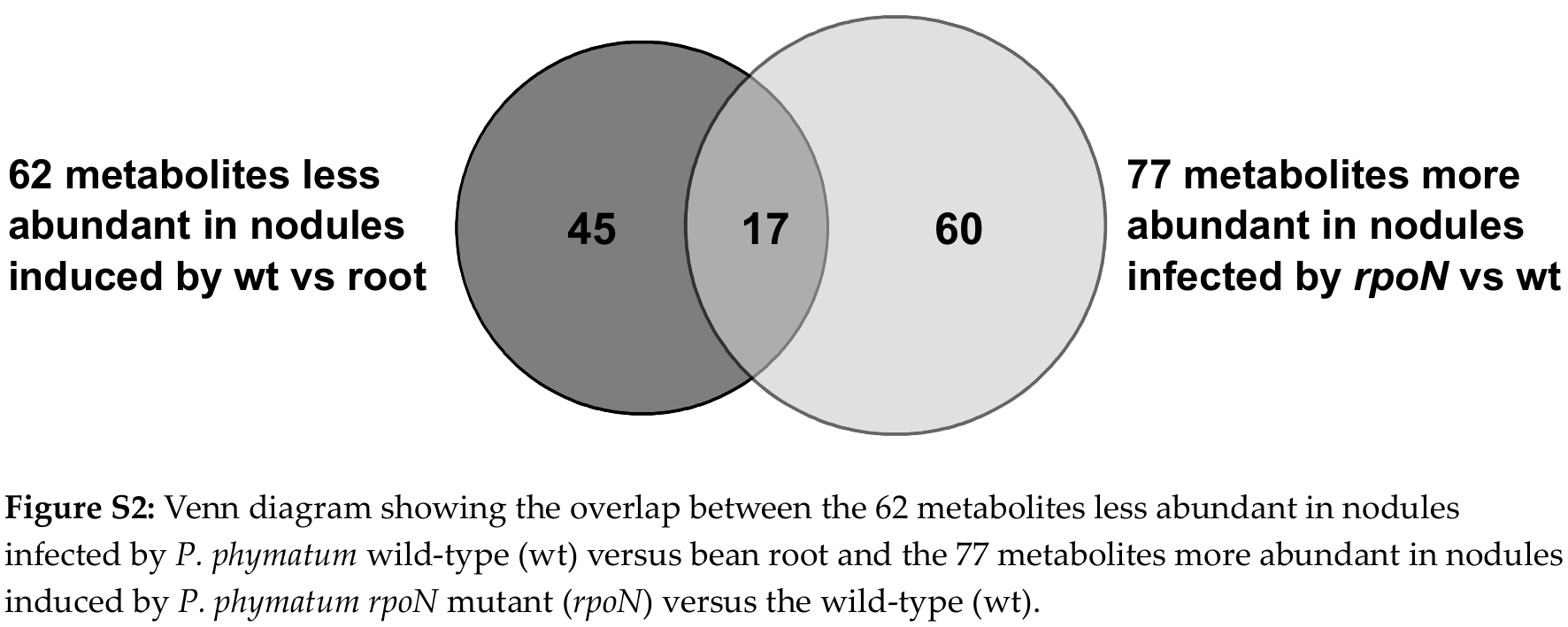

Supplement: Supplementary file 1 [file ijms-19-01049-s001.zip › Supplementary files_images+tables/Figure S2_20180326.tiff]

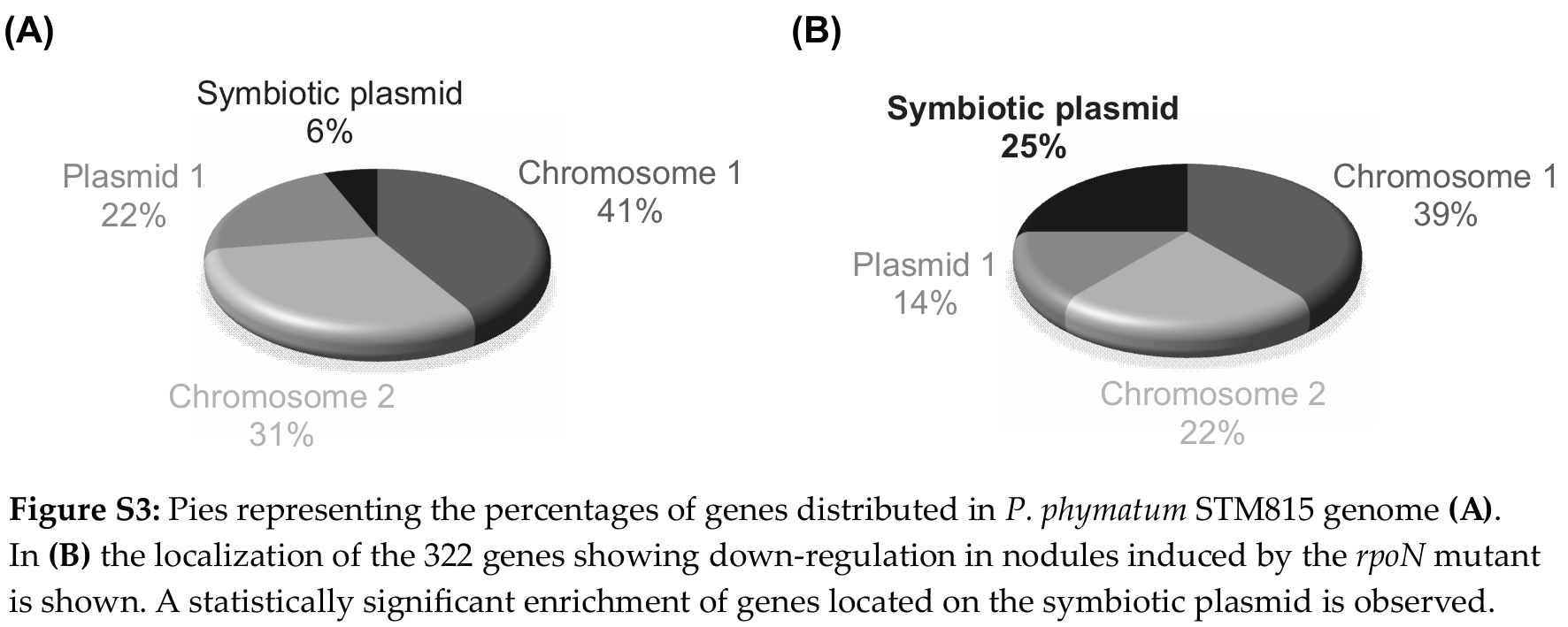

Supplement: Supplementary file 1 [file ijms-19-01049-s001.zip › Supplementary files_images+tables/Figure S3_20180326.tiff]

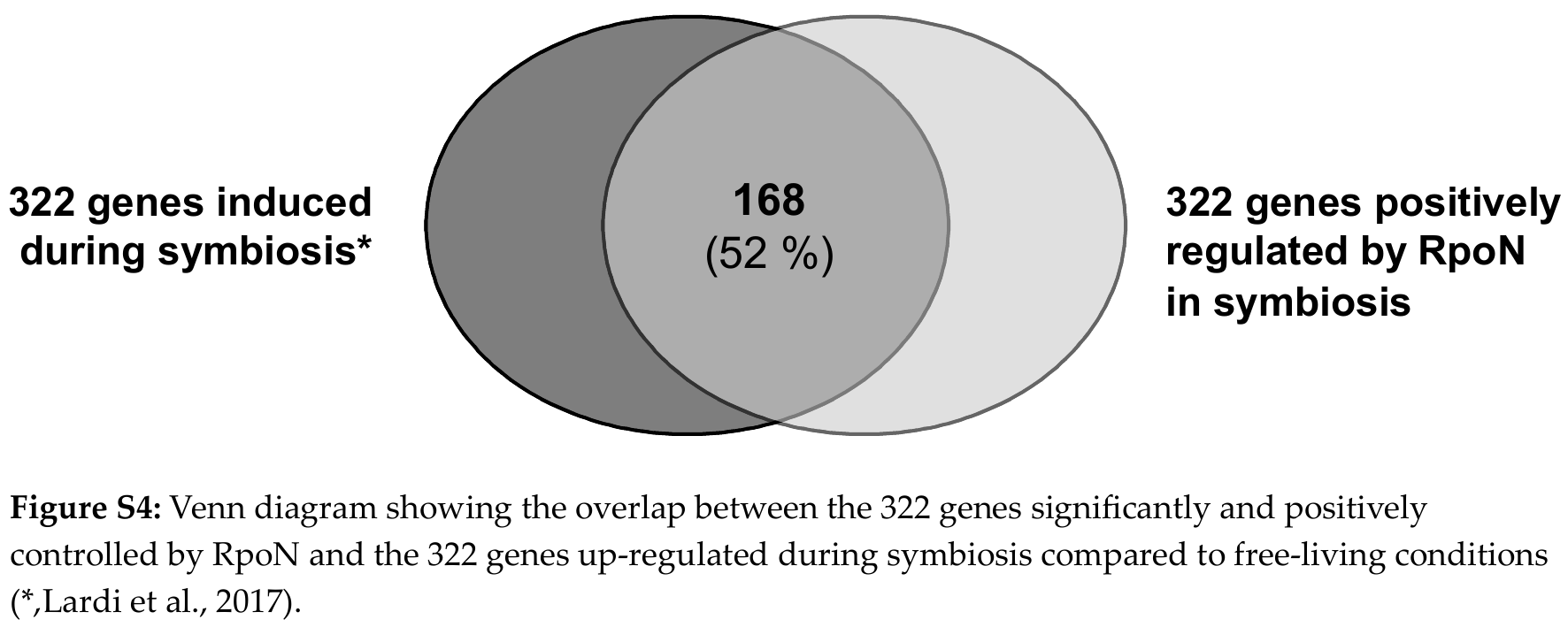

Supplement: Supplementary file 1 [file ijms-19-01049-s001.zip › Supplementary files_images+tables/Figure S4_20180326.tiff]

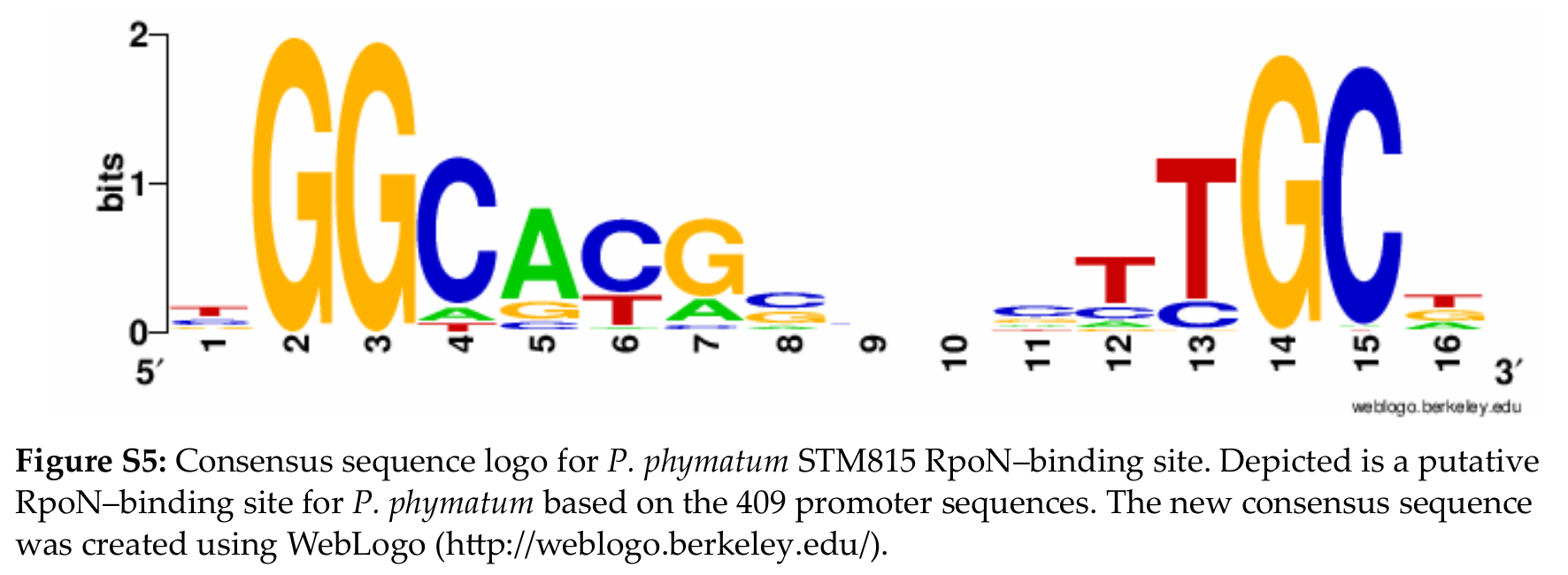

Supplement: Supplementary file 1 [file ijms-19-01049-s001.zip › Supplementary files_images+tables/Figure S5_20180326.tif]
